# Modeling the effect of imported malaria on the elimination programme in KwaZulu-Natal province of South Africa

**DOI:** 10.11604/pamj.2024.47.80.35882

**Published:** 2024-02-21

**Authors:** Peter Joseph Witbooi, Gbenga Jacob Abiodun, Rajendra Maharaj

**Affiliations:** 1 Department of Mathematics and Applied Mathematics, University of the Western Cape, Private Bag X17, Bellville 7535, South Africa,; 2 Department of Mathematics, Southern Methodist University, Dallas, TX 75275, USA,; 3 Office of Malaria Research, South African Medical Research Council, Durban, South Africa,; 4 School of Life Sciences, College of Agriculture, Engineering and Sciences, University of KwaZulu-Natal, South Africa

**Keywords:** Epidemiological models, Culicidae, malaria, prevalence, transients, migrants, certification, South Africa, Mozambique, World Health Organization

## Abstract

**Introduction:**

with imported malaria cases in a given population, the question arises as to what extent the local cases are a consequence of the imports or not. We perform a modeling analysis for a specific area, in a region aspiring for malaria-free status.

**Methods:**

data on malaria cases over ten years is subjected to a compartmental model which is assumed to be operating close to the equilibrium state. Two of the parameters of the model are fitted to the decadal data. The other parameters in the model are sourced from the literature. The model is utilized to simulate the malaria prevalence with or without imported cases.

**Results:**

in any given year the annual average of 460 imported cases, resulted in an end-of-year season malaria prevalence of 257 local active infectious cases, whereas without the imports the malaria prevalence at the end of the season would have been fewer than 10 active infectious cases. We calculate the numerical value of the basic reproduction number for the model, which reveals the extent to which the disease is being eliminated from the population or not.

**Conclusion:**

without the imported cases, over the ten seasons of malaria, 2008-2018, the KwaZulu-Natal province would have been malaria-free over at least the last 7 years of the decade indicated. This simple methodology works well even in situations where data is limited.

## Introduction

Although Africa is the continent with the highest burden of malaria morbidity and mortality, great strides have been made in drastically reducing cases and deaths (WHO 2019). Many countries on the continent are now targeting malaria elimination as a result of this reduced burden of disease. The southern African region is leading the way with all eight southern African countries targeting malaria elimination by 2030. This ideal is the aim of the Elimination 8 Initiative which is strengthening the capacity of the eight countries falling under its umbrella to control and eventually eliminate malaria from the region. However, in the last five years progress towards elimination has stalled and even reversed in some countries. The Elimination 8 countries are back on track with their elimination agendas through coordinated action in vector control and case management.

As one of the Elimination 8 countries, South Africa is targeting malaria elimination by 2023. The three endemic provinces are at different stages of the WHO malaria elimination spectrum with Limpopo province still in the control phase whilst Mpumalanga is in the pre-elimination phase. KwaZulu-Natal (KZN) is the only province that is firmly in the elimination phase having shown a steady decline in the number of local cases reported in the past twelve years. Historically, this province was the main contributor to the malaria burden of the country, especially in the epidemic of 1999 - 2000 when KZN accounted for two-thirds of the cases that were reported from the country during that malaria season. At that stage, the main drivers of the high transmission levels were vector resistance to the pyrethroid insecticides used in the control programme and parasite resistance to the first-line treatment of choice, sulphadoxine-pyrimethamine. Through the reintroduction of DDT to replace the pyrethroid insecticides, vector populations were rapidly brought under check thus flattening the epidemiological curve. Transmission was further curtailed through the introduction of artemisinin combination therapy, thus moving away from reliance on a single drug to control the parasites in the human hosts.

Although the local transmission of cases has reduced to less than 100 cases in the past five years, very low levels of transmission persist, being attributed to imported malaria with secondary transmission of the disease. The study [1] by Raman *et al*. (2020) found that the importation of malaria via asymptomatic individuals from Mozambique was driving the low-level residual malaria reported from the province. To meet the elimination goals, a foci-clearing programme was adopted. However, imported malaria kept influencing the delineation of the foci. It was found that the influx of malaria-infected individuals into a population protected by a coordinated and sustained control programme in which there is successful control poses a major problem. People in controlled areas would have less immunity to the disease than people receiving many infected bites which develop their immunity. The majority of migrants originate from Mozambique and to a lesser extent Zimbabwe. Movement of people occurs in large numbers across the formal border crossings and there is greater movement of people through informal border crossings [1]. Due to the volume of traffic and the time taken to test and treat, border screening is not possible as it is also labor-intensive and cannot be consistently maintained. Thus, the movement of people from areas of high transmission in southern Mozambique into the KwaZulu-Natal (KZN) province of South Africa, poses a continuing risk, especially to border populations. For South Africa to reach elimination and ultimately eradication of malaria, the movement of infected people across international boundaries needs to be curbed. This study was conducted to model the impact of imported malaria on the elimination agenda in KZN.

In this paper, a modeling analysis is performed on the effect of imported malaria cases on a population where malaria is systematically being controlled toward elimination. Data on malaria cases over ten years is subjected to a compartmental model of ordinary differential equations (ODEs). The data enables us to calculate the numerical values of crucial parameters. After that, we remove the effect of the imported cases. Thus we can determine the extent to which the disease has (or could have) been eliminated from the population.

A study [2] on malaria elimination progress has found that over the decade 2010 -2019, a considerable proportion of malaria cases in KZN was linked to traveling in malaria-endemic areas outside of KZN. The current method can quantify the effect to which the KZN local cases are a consequence of the imported cases. Work on the elimination of malaria in the Mpumalanga province of South Africa also appears in the work [3] of Silal *et al*. (2014), using a compartmental model with 9 classes which is very different from the popular ODEs model structure. A related theme is studied in [4] (Yacheur *et al*. 2019), but with a completely different model of 11 compartments versus the 7 compartments of our model.

## Methods

In this paper, malaria prevalence numbers for KZN over the period 2008 -2018 as reflected in Maharaj *et al*. [5], are converted to 2018 present values, with respect to Republic of South Africa (RSA) population growth rate. These values for the 10 seasons are then averaged. The information is fed into a mathematical compartmental model [6] which enables us to calculate the biting rate and the disease induced mortality rate. The latter information can now be utilized to reveal the progression of malaria in the population assuming that there are no imported cases.

**Figure 1:**
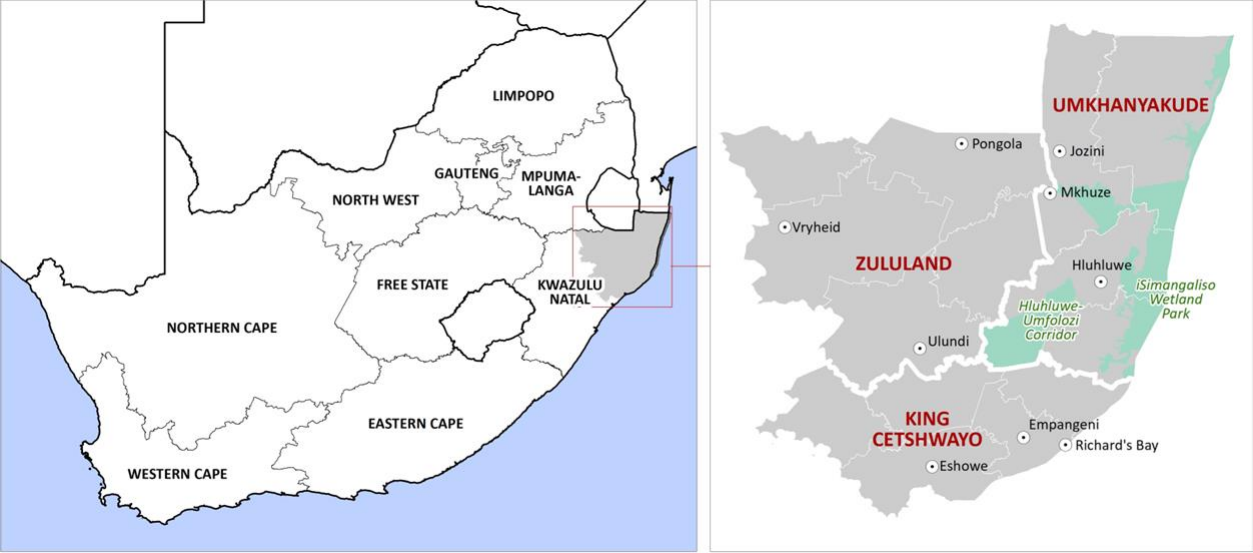
the study region comprises the three districts in KwaZulu-Natal as depicted on the enlarged map on the right-hand side

**Study area:** the malaria region of KZN, included within the districts of uMkhanyakude, King Cetshwayo, and Zululand (Figure 1). This region carries a population of more than 2.6 million (Table 1) at risk of malaria.

**Table 1:** parameters, description and numerical values (per day, where relevant)

Symbol	Description	Numerical value
*μ*	Mortality rate for humans, not including death directly due to malaria	4.657/10000 (per day)
*θ*	Mortality rate of vectors	0.04
*b*	The probability that a bite by an infected mosquito will lead to a (new) human infection	0.075
*c*	The probability that a bite on an infected human will lead to a (new) mosquito infection	0.0375
*ρ_h_*	Transfer rate of humans from *E_h_* class to *I_h_*-class	1/12
*σ*	Transfer rate of humans from class *I_h_* to *R*-class (recovery rate)	1/180
*ζ*	Transfer rate from *R*-class to *S*-class (rate of loss of temporary immunity)	1/730
*ρ_v_*	Transfer rate of vectors from *E_v_* -class to *I_v_*-class	1/8
*γ_1_*	Mortality rate due to IRS in class	0.004
*γ_2_*	Mortality rate due to IRS in class	0.004
*K_0_*	Human birth rate	122.498
L	Mosquito birth rate	2,496 000
*K_1_*	Rate of inflow of latently infected humans	1.9167
*K_3_*	Rate of inflow of infectious humans	0
*K_3_*	Rate of outflow of recovered migrants	1.87719
*α*	The probability of a specific human getting bitten by a mosquito during a one-day period	1.0011x10^-8^
*δ*	The rate of human deaths due to malaria	0.0000672

**Data:** essentially, the data used in this paper are the malaria infection cases and mortalities in KZN over the period 2008 - 2018, as reported in [5]. Population growth figures are obtained from Worldometers [7].

**Figure 2:**
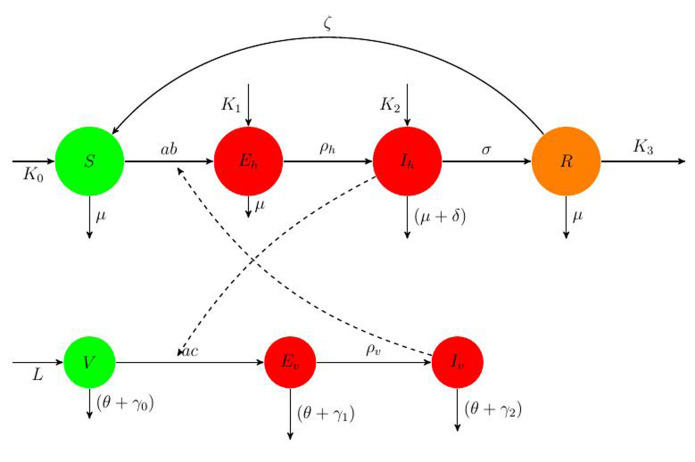
a flow chart depicting the population dynamics of malaria; the broken arrows denote action on the target flow, not flow of individual humans or vectors

**Mathematical model:** we utilize the compartmental model from [6], of ordinary differential equations to investigate and quantify the number of malaria infection cases in KZN, together with the effect of infected migrants from elsewhere moving in and out of KZN. The model accommodates the in-door residual spraying (IRS) intervention and allows for (sporadic) influx of infected humans. We note that infected mosquitos from outside the borders do not penetrate very far into the area on their own but they can be carried by taxis and other road transport in small numbers. For the purpose of the current study, we assume that the effect of imported infected mosquitos on the total human population is negligible. In the current paper we apply the model [6] in producing computations and simulations as an application, in order to further expound on the research in [1,5,8]. A flow chart for the model is shown in Figure 2 and the parameters of the ODEs are described in Table 1. Also in Table 1, we declare the numerical values of the parameters. As in the model in [6], we partition the human population into four classes: susceptibles (S), latent-infected (*E_h_*), infectious (*I_h_*) and recovered (R), while the vector population is divided into three classes: the susceptibles (V), latently infected (*E_v_*) and infectious (*I_v_*). Both the human and vector populations are assumed to be homogeneously mixing. Then the model comprises the following system of ODEs. The system of ordinary differential equations:


$$\dot{S}=K_{0}+\zeta R-abI_{v}S-\mu S$$

$${\dot{E}}_{h}=K_{1}+abI_{v}S-\left(\rho_{h}+\mu \right)E_{h}$$

$${\dot{I}}_{h}=K_{2}+\rho_{h}E_{h}-\left(\mu +\delta +\sigma \right)I_{h}$$

$$\dot{R}=\rho I_{h}-\left(\mu +\zeta \right)R-K_{3}$$

$$\dot{V}=L-acI_{h}V-\left(\theta +\gamma_{0}\right)V$$

$${\dot{E}}_{v}=acI_{h}V-\left(\theta +\rho_{v}+\gamma_{1}\right)E_{v}$$

$${\dot{I}}_{v}=\rho_{v}E_{v}-\left(0+\gamma_{2}\right)I_{v}$$

$$\text{with }K_{3}=\sigma \left[K_{2}+\frac{\rho_{h}K_{1}}{\mu +\rho_{h}}\right]/\left(\mu +\delta +\sigma \right).$$

We choose the parameters for the model to apply as best as possible to KZN. Most of the parameters are of biological type and will be sourced from relevant papers, essentially [9] and sources quoted therein. The incubation period of *Plasmodium falciparum* is 9-14 days in humans [10] and 7-9 days in mosquito vectors [11]. Based on these numbers, we choose the relevant parameter values *ρ_h_*= 1/12 per day and *ρ_v_*= 1/8 per day. The parameters *γ_1_* and *γ_2_* will be assigned nominal values since the (quantitative) effect of IRS on vector mortality in the region is not well documented.

Parameters relating to population sizes have to be carefully calculated. It is common for ODE compartmental models to assume that there is no long-term population growth. Minor temporary fluctuations in the population size may arise from disease-induced mortalities. Our model is also based on this assumption. For this reason, we adapt the numerical values of parameters related to the human population such as to be relevant for the year 2018. This means that population data will be converted to their 2018-equivalent values. These converted numbers are calculated with respect to the population growth of RSA as reflected in [7]. The vector population is estimated at approximately 20 times the size of the human population as in [6].

The total population at risk is obtained from the document [12], and this determines the parameter *K_0_*. We assume the average population density of the vectors over the region to be 20 times the population density of humans. The parameters *K_1_* and *K_2_* are obtained by calculating the mean per day of the (2018-converted values) of the imported cases as in [5]. So we obtain *K_1_+K_2_*, and we have to split this value between *K_1_* and *K_2_*. However, the split between *K_1_* and *K_2_* is difficult to determine, and we shall just make a nominal split. Our fitted rates a and *δ* stay the same up to 3 significant digits irrespective of the split. The parameters *μ, θ, b, c, σ, ζ* are deduced from (respectively) the sources [9,13-18].

Two of the parameters of our model will be fitted from [5]. These two are the biting rate a and the malaria-induced mortality rate *δ*. We can assume that most of the malaria-infected migrants are in the latent phase. From [5] we calculate the mean number of malaria mortalities per day over the ten years and the mean value per day of the total number of malaria infections. These latter two values are used together with the model system of ODE, to simultaneously calculate the equilibrium state and the values of the parameters a and *δ*. In the case of no influx of humans into the population, the model has a disease-free equilibrium point *X_0_= (K_0_/μ, 0, 0, 0, L/(θ+γ_0_), 0, 0)* and a basic reproduction number *R_0_*:


$$R_{0}=\sqrt{\frac{a^{2}bc\rho_{h}\rho_{v}K_{0}L}{\mu \left(\mu +\rho_{h}\right)\left(\mu +\delta +\sigma \right)\left(\theta +\gamma_{0}\right)\left(\theta +\rho_{v}+\gamma_{1}\right)\left(\theta +\gamma_{2}\right)}}$$

From [6], it can be deduced that if *R_0_*< 1, then the disease-free equilibrium is globally asymptotically stable. This means that if there is zero influx of imported cases and *R_0_*< 1, then given any initial state of the population, the disease will eventually vanish from the population, that is, malaria will be eliminated from that population. The model is run on data from [5] and parameter values as in Table 1. Different scenarios of influx are considered.

## Results

From the paper [5], for the 10 years 2008-2018, we calculated the 2018-equivalent values of the relevant malaria incidence data, using general population numbers for South Africa from [7]. The following values were calculated. The mean annual number of imported malaria cases: 460; the mean annual number of malaria cases both local and imported infections: is 534; and the mean annual number of malaria deaths: is 6.32. This enabled us to calculate the two unknown parameters, i.e, the biting rate *α* and the malaria-induced mortality rate *δ*, and thus the model is completely calibrated. The incubation period of *Plasmodium falciparum* is 9-14 days in humans [10] and 7-9 days in mosquito vectors [11]. Based on these numbers, we choose the relevant parameter values *ρ_h_*= 1/12 per day and *ρ_v_*= 1/8 per day.

The population at risk is obtained from [12] as having the numerical value 2 630 100, and this corresponds to *K_0_/μ*. The complete set of parameter values is listed in Table 1. A key result is that for the special scenario of having no influx of infected individuals, we can now calculate the basic reproduction number *R_0_*= 0.3703. The equilibrium values of the various compartments, also obtained from the given parameter values, are as follows:


$$S*=2.617e+006,E_{h}^{*}=17.50,I_{h}^{*}=257.3,R*=119.2,V*=6.240e+007,E_{v}^{*}=35.66,I_{h}^{*}=101.3.$$

**Figure 3:**
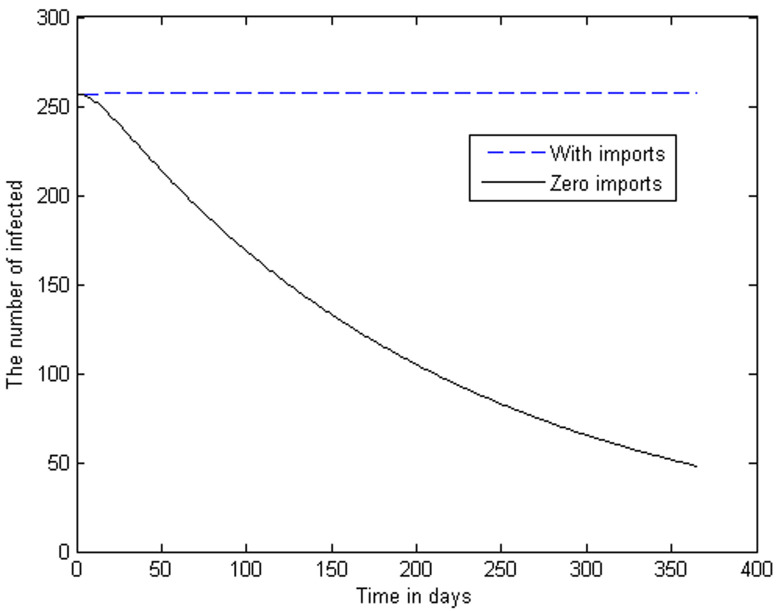
trajectories of the active infected human classes, showing the reduction in the number of active malaria cases when there are zero imported cases

**Figure 4:**
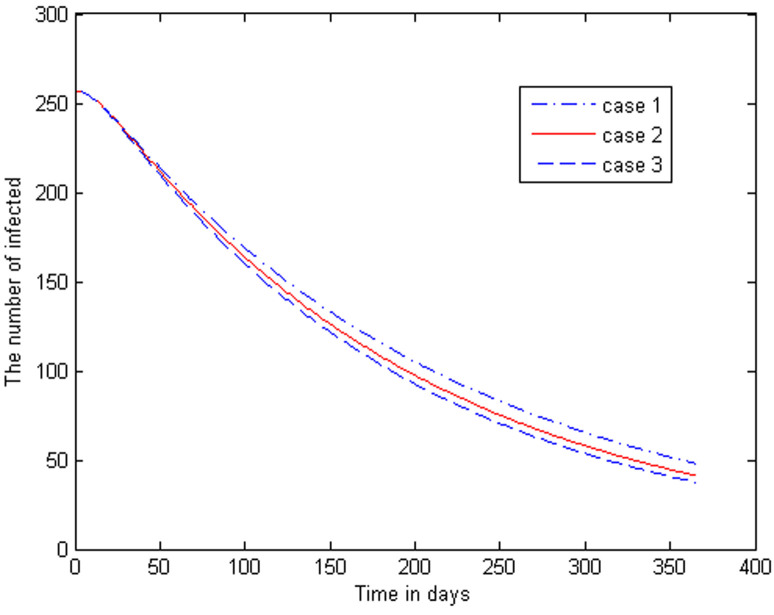
trajectories of the infectious human class for different levels of insecticide induced mortality rates

Now we can produce a range of graphs, using the given parameters and variations on these parameters. The two curves in Figure 3 represent the *I_v_*-class (i.e. active malaria cases) for two different scenarios. The higher curve indicates the steady state when we have an inflow of infected humans at the constant average rate calculated from [5]. The decreasing curve shows the phenomenal reduction in the number of active malaria cases when there are zero imported cases. In Figure 4, the trajectories of the infectious human class are constructed for the case that the insecticide-induced mortality rates in the infectious and latent classes of the vector are intensified to levels higher than the assumed baseline levels. The following cases are considered: Case 1 is the output of the model with parameter values as in Table 1. Case 2 is the output with the parameters γ_1= 0.75θ and γ_2= 0.75θ. Case 3 is the output with γ_1= 1.5θ and γ_2=1.5θ.

**Figure 5:**
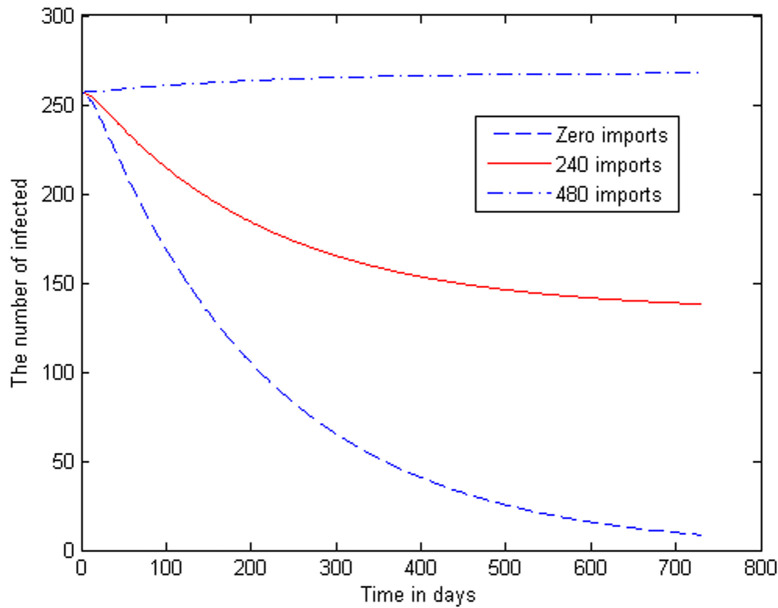
trajectories of the infectious human class for different rates of inflow of infected humans

In Figure 5 we run the model with different levels of imported malaria cases, over 2 years. We obtain three curves, considering different levels of inflow of latently infected humans into the KZN population, these levels being 480 p.a., 240 p.a., and also zero p.a. More precisely, the curves correspond to the following levels of inflow of infected: Case 1 is the output of the model with zero imported malaria cases. Case 2 is the output with 240 imported cases per year. Case 3 is the output with 480 imported cases per year.

## Discussion

The model quantifies the effect of infected immigrants. Even with *R_0_*= 0.7405 < 1 as computed, it can be observed that due to the influx of infected individuals, the infection stays in the population. On the other hand, noting that *R_0_* is less than unity, the disease will indeed vanish from this population if there is no inflow of infectious humans. Figure 3 shows how infections fall over 1 year if the inflow of infected individuals is zero. Even if the biting rate would have been double the value in Table 1 i.e., if we had taken α=2 x 1.0011 x10^-8^, then we would have had *R_0_*= 0.7405, which is still less than 1, guaranteeing the ultimate elimination of the disease. The graph in Figure 3 shows how the class of infectious individuals decreases sharply if we start at the equilibrium state calculated above, and we keep the inflow of infected at zero.

Regarding IRS we make the following observations. If the insecticide-induced mortality rate is higher, we would expect the human infectious class to be higher than the infectious class in the case of a lower insecticide-induced mortality rate. The graphs in Figure 4 reveal that there is a notable decrease in malaria prevalence whenever the IRS is intensified. As in the standard case, we observe a very significant decline in the infectious class. The lowest curve corresponds to an insecticide-induced mortality rate which is 1.5 times the general mortality rate. We expect this to lead to a faster extinction rate of malaria, as is indeed shown in the graph.

In Figure 5 we run the model with different levels of imported malaria cases, over 2 years. The curve showing the highest malaria prevalence is due to an influx of 480 cases per year. In this case, the influx is higher than the (population growth rate adapted) average influx over the decade. Consequently, it leads to a malaria prevalence that is higher than the initial value. Then we did it for 240 imported cases per year and we observed that it also gives a fairly high malaria prevalence over the 2 years, with active cases standing at 138 at the end of the 2 years. In the third case, we have done the *I(t)*-trajectory for the case of zero imported malaria cases. In this case, we notice how the malaria prevalence falls over the two years, with active cases dropping from 257 to 9. We have demonstrated the disturbing effect of imported malaria into a population which would otherwise have moved towards zero prevalence.

**Study limitations:** while malaria transmission is affected by climatic conditions and ideally should be modelled as such, it is commonly modelled without accommodating climatic variables. The latter approach was followed in the current analysis. The results can be improved by using (far more complex) climatic models and climatic data.

## Conclusion

The analysis in this study shows the importance of a good understanding of the impact of imported cases into a population that is at risk of malaria. This methodology can be utilized more generally. A study in this regard has already been conducted on the effect of imported measles on a local population [19]. We note the huge spike in malaria cases in South Africa during the period 1996-2001 [20]. The paper [6] investigates the role of the importation of cases in the latter event. Migration of vectors over borders can be an additional cause of infections into the population in point. One can readily modify the mathematical model to accommodate the influx of infected mosquitoes, but the rate of immigration of mosquitos is difficult to quantify. Nevertheless, near the Mozambique border, the human population is sparse, and the effect of such an influx of vectors is not as severe as it would have been otherwise.

The modeling was performed assuming that the state of the disease in the population is near to equilibrium, and by taking the annual average per season over 10 seasons. This is a good approximation with a fairly clear answer. More accurate analysis can be done for a specific season if the initial prevalence numbers of malaria cases in the population are known. Also, a climate-based model (e.g., [13,21]) will ensure better versatility and accuracy. Nevertheless, the current methodology works well when data is limited. The modeling shows that over the ten seasons of the period 2008-2018, the KZN province would have been very close to malaria-free if the imported cases could have been avoided. The results demonstrate that KZN would have become malaria-free very quickly after the termination of imported cases. This makes the case for sub-national elimination certification; the imported cases need to be prevented as a measure to prevent the reintroduction of the disease into areas where the disease has been eliminated.

### What is known about this topic

- *During the 1990, the KZN province of South Africa contributed approximately two-thirds of the malaria cases of South Africa, but in KZN the numbers have significantly dropped over two decades since the year 2000, even to the extent that KZN is now leading the way to elimination of malaria;*
- *Over the years 2008 -2018 a significant fraction of malaria cases in KZN province of South Africa has been found to be imported cases*.

### What this study adds

- *This study quantifies the extent to which imported malaria cases contribute to local transmission in KZN;*
- *It is revealed that malaria transmission would have consistently decreased in KZN and exactly how close to elimination KZN would have been if the imported cases could have been avoided*.
